# The Significance of Sensitive Interferon Gamma Release Assays for Diagnosis of Latent Tuberculosis Infection in Patients Receiving Tumor Necrosis Factor-α Antagonist Therapy

**DOI:** 10.1371/journal.pone.0141033

**Published:** 2015-10-16

**Authors:** Yu Jung Jung, Hye In Woo, Kyeongman Jeon, Won-Jung Koh, Dong Kyoung Jang, Hoon Suk Cha, Eun Mi Koh, Nam Yong Lee, Eun-Suk Kang

**Affiliations:** 1 Departments of Laboratory Medicine and Genetics, Samsung Medical Center, Sungkyunkwan University School of Medicine, Seoul, Korea; 2 Department of Laboratory Medicine, Samsung Changwon Hospital, Sungkyunkwan University School of Medicine, Changwon, Korea; 3 Division of Pulmonary and Clinical Care Medicine, Department of Medicine, Samsung Medical Center, Sungkyunkwan University School of Medicine, Seoul, Korea; 4 Department of Gastroenterology, Samsung Medical Center, Sungkyunkwan University School of Medicine, Seoul, Korea; 5 Division of Rheumatology, Department of Medicine, Samsung Medical Center, Sungkyunkwan University School of Medicine, Seoul, Korea; Hospital San Agustín. Aviles. Asturias. Spain, SPAIN

## Abstract

**Objective:**

We compared two interferon gamma release assays (IGRAs), QuantiFERON-TB Gold In-Tube (QFT-GIT) and T-SPOT.TB, for diagnosis of latent tuberculosis infection (LTBI) in patients before and while receiving tumor necrosis factor (TNF)-α antagonist therapy. This study evaluated the significance of sensitive IGRAs for LTBI screening and monitoring.

**Methods:**

Before starting TNF-α antagonist therapy, 156 consecutive patients with rheumatic diseases were screened for LTBI using QFT-GIT and T-SPOT.TB tests. According to our study protocol, QFT-GIT-positive patients received LTBI treatment. Patients positive by any IGRAs were subjected to follow-up IGRA tests after completing LTBI-treatment and/or during TNF-α antagonist therapy.

**Results:**

At the initial LTBI screening, 45 (28.9%) and 70 (44.9%) patients were positive by QFT-GIT and T-SPOT.TB, respectively. The agreement rate between IGRA results was 78.8% (k = 0.56; 95% confidence interval [95% CI] = 0.43 to 0.68). Of 29 patients who were positive only by T-SPOT.TB in the initial screening, 83% (19/23) were persistently positive by T-SPOT.TB, while QFT-GIT testing showed that 36% (9/25) had conversion during TNF-α antagonist therapy. By the end of the follow-up period (218 to 1,264 days), four patients (4/137, 2.9%) developed active tuberculosis (TB) diseases during receiving TNF-α antagonist therapy. Among them, one was Q-T+, one was Q+T-, and the remaining two were Q-T- at the initial screening (Q, QuantiFERON-TB Gold In-Tube; T, T-SPOT.TB; +, positive; -, negative). Two (2/4, 50%) patients with TB reactivation had at least one prior risk factor consistent with previous TB infection.

**Conclusion:**

This study demonstrated the need to capitalize on sensitive IGRAs to monitor for LTBI in at-risk patients for a more sensitive diagnosis in countries with an intermediate TB burden.

## Introduction

Reactivation of latent tuberculosis infection (LTBI) is one of the major complications of tumor necrosis factor (TNF)-α antagonist therapy in patients with rheumatic diseases [1, 2]. The chance of reactivation may increase with certain medical conditions such as human immunodeficiency virus (HIV) infection and concurrent medication including immunosuppressive drugs [3, 4], which are administered to most patients with rheumatic diseases. Therefore, to avoid these possible side effects, testing should be performed prior to initiating TNF-α antagonist therapy. Traditionally, LTBI screening is conducted through tuberculin skin tests (TSTs) before administering immunosuppressive agents. However, in patients who have received Bacillus Calmette Guérin (BCG) vaccinations, TST is not optimal since this test has well-known sensitivity and specificity limitations [5, 6] that could result in false-positive results [7].

There have been extensive efforts to develop better tools for detection of LTBI in patients with rheumatic diseases who require immunosuppressive agents such as TNF-α antagonist. Interferon-gamma release assays (IGRAs) such as QuantiFERON-TB Gold In-Tube (QFT-GIT, Cellestis/Qiagen, Carnegie, Australia) and T-SPOT.TB (Oxford Immunotec, Abingdon, UK), which use different detection principles from TST, have been developed as TST complements or replacements. However, there is no consensus on currently available IGRA utilization for diagnosis of LTBI, because IGRA performance varies according to study group and design.

Several studies [8–11] have serially monitored IGRA results in patients with rheumatic diseases receiving TNF-α antagonist therapy, but there are no standard recommendations for the timing of follow-up LTBI testing after the initial screening, how to interpret test results, and which patients should receive treatment based on subsequent results.

In this study, we aimed to elucidate an effective diagnostic approach for initial diagnosis and monitoring of LTBI and the significance of follow-up tests for LTBI detection using QFT-GIT and T-SPOT.TB tests in patients administered TNF-α antagonist therapy.

## Materials and Methods

### Patients

This study was approved by the Institutional Review Board of the Samsung Medical Center (approval number 2009-06-076). This study was conducted according to principles in the Declaration of Helsinki. All patients agreed and provided written informed consent for participation in this study.

We performed a prospective study on patients with various rheumatic diseases to screen for LTBI before administering TNF-α antagonist therapy. A total of 156 consecutive patients were enrolled between July 2009 and January 2012. Routine approaches for LTBI diagnosis including TST, chest radiography, and QFT-GIT testing were performed in all patients. T-SPOT.TB tests were performed in parallel only for comparison with QFT-GIT results. Patients who tested positive by any IGRA method at the initial screening were subjected to follow-up IGRA tests at the end of LTBI treatment and/or while receiving TNF-α antagonist therapy. Except for the baseline QFT-GIT testing, all other tests performed during the follow-up period were solely for research purposes since QFT-GIT was the only Korea Food and Drug Administration-approved IGRA at the time. Our treatment protocol [12, 13] called for treatment of patients positive by QFT-GIT. TSTs were used to clarify indeterminate QFT-GIT results. Positive tests were defined according to the Korean National Guidelines [14, 15] as induration of 10 mm after 48–72 hours. Patients with TST results greater than or equal to 10 mm and indeterminate QFT-GIT results received LTBI treatment.

### IGRAs: QFT-GIT and T-SPOT.TB

Both IGRA tests were performed according to the manufacturer’s instructions and interpreted by the recent Centers for Disease Control and Prevention (CDC) criteria [16].

QFT-GIT is an enzyme linked immunosorbent assay (ELISA) based test that uses peptide mixtures that stimulates three *M*.*tuberculosis* antigens, including early secretory antigenic target-6 (ESAT-6) and culture filtrate protein 10 (CFP-10), which are encoded in the region of difference (RD) 1, and TB7.7, encoded by the RD 11 region. A total of three tubes including positive control (mitogen), negative control (saline), and TB-specific antigens (ESAT-6, CFP-10, and TB 7.7 in a single tube) with 1 mL heparinized whole blood were prepared and incubated at 37°C for 16–24 hours. Plasma harvested from each tube after incubation was tested for IFN-γ by ELISA. The levels of IFN-γ (IU/mL) were derived from a standard curve. The cutoff IFN-γ response value was 0.35 IU/mL, which was calculated by subtracting the negative control value from that of the TB antigen-stimulated sample only when the negative control was below 8.0 IU/mL. If an IFN-γ response in TB antigen-stimulated tube was less than 25% of the negative control, it was considered negative even though the subtracted value was more than 0.35 IU/mL. Test results with a negative control over 8.0 IU/mL or a positive control less than 0.5 IU/mL were considered indeterminate.

T-SPOT.TB is a simplified enzyme-linked immunospot (ELISPOT) assay that uses two TB-specific antigens, ESAT-6 and CFP10. Peripheral blood mononuclear cells are first separated from heparinized whole blood samples by density gradient separation and stimulated with each of the positive control (phytohaemagglutinin), negative control (saline), as well as ESAT-6 and CFP10 in microplate wells coated with IFN-γ capture antibodies. After 16–20 hours of incubation and wash, individual activated cells in each sample were enumerated as distinct dark blue spots positive for IFN-γ antibodies. The results were interpreted by subtracting the spot count in the negative control from ESAT-6 and CFP10 counts. The results were classified as positive, negative or indeterminate. Samples with six or more spots with negative controls containing 0–5 spot-forming cells (SFCs) were considered positive. For negative controls containing 6–10 SFCs, positive patient samples had twofold greater SFCs. Subtracted spot counts of five or less were considered negative. Indeterminate test results were defined as the presence of more than 10 SFCs in the negative control and/or less than 20 SFCs in the positive control. As proposed in a previous study [17], borderline zones were defined as 0.35–0.7 IU/mL in QFT-GIT and 6–8 SFCs in T-SPOT.TB.

IGRA test conversion was defined a change to a positive result in patients negative at baseline, and vice versa for reversion.

### Statistical analysis

Continuous variables were expressed as medians, and interquartile range and nominal variables were presented as absolute and percentage values (%). Two-sided Fisher’s exact test and Mann-Whitney test were used for categorical variables and continuous variables, respectively. The concordance of QFT-GIT and T-SPOT.TB results was assessed using Cohen’s kappa test (κ-value > 0.75, excellent agreement; 0.40 to 0.75, fair to good agreement; and < 0.40, poor agreement). When we calculated agreement, we regarded the indeterminate results as negative. MedCalc version 10.2.0.0 (MedCalc Software BVBA, Ostend, Belgium) was used for statistical analyses. *P* values less than 0.05 were considered statistically significant.

## Results

### Baseline patient characteristics

The demographic and initial clinical characteristics of all 156 patients are shown in Table 1. There were 96 (61.5%) males and 60 (38.5%) females, with a median age of 42 years (range: 17 to 73 years). Rheumatoid arthritis and ankylosing spondylitis comprised 48% (75/156) and 46% (71/156) of the patient population, respectively. Of 156 patients, seven had a history of TB exposure, and 17 had received previous TB treatment. Eight patients (5.1%) had abnormal chest radiography findings such as nodular or fibrotic lesions consistent with previous tuberculosis (TB) infection. The TST-positive rate with induration diameters greater than 10 mm was 43% (64/150). Fifty-five percent (86/156) of all patients were receiving at least one concurrent immunosuppressive drug at the time of enrollment. A total of 137 patients with or without LTBI treatment were administered TNF-α antagonist therapy after LTBI screening, 27 of which had been administered two or more TNF-α antagonist agents.

**Table 1:** Patient characteristics.

Characteristics	Total
Number of patients	156
Age, years	42 (32–54)
Number of patients >50years	46 (29)
Number of male patients	96 (62)
Diagnosis	
Rheumatoid arthritis	75 (48)
Ankylosing spondylitis	71 (46)
Others^a^	10 (6.4)
History of previous TB exposure (+)	7 (4.5)
History of previous TB treatment (+)	17 (11)
Tuberculin skin test (+)	64 (43)^b^
Chest radiography (previous TB +)	8 (5.1)
TNF-α antagonist^c^	
Etanercept	79 (58)
Adalimumab	65 (47)
Infliximab	21 (15)
Concurrent immunosuppressive therapy	
Methotrexate	71 (46)
Tacrolimus	3 (1.9)
Steroid	74 (47.4)

The data are presented as median and interquartile range or as number (%).

TB, tuberculosis; +, positive; TNF, tumor necrosis factor.

^a^Inflammatory bowel disease (n = 5; 2 with ulcerative colitis and 3 with Crohn’s disease), spondyloarthropathy (n = 2), and psoriatic arthropathy (n = 3)

^b^Of 156 patients included in this study, 150 were tested by tuberculin skin test.

^c^A total of 137 patients received TNF-α antagonist therapy. Twenty-seven patients were administered two or more drugs.

### Detection of LTBI by two IGRAs

Forty-five (28.9%) and 70 (44.9%) patients were positive by QFT-GIT and T-SPOT.TB, respectively. Among the 45 QFT-GIT-positive patients, four were T-SPOT.TB-negative. However, of 70 T-SPOT.TB-positive patients, 28 were negative and one was indeterminate by QFT-GIT. Three (1.9%) and six (3.8%) patients had indeterminate results by QFT-GIT and T-SPOT.TB, respectively. A moderate level of agreement (78.8%) was found between two IGRAs in this study (k = 0.56; 95% confidence interval [95% CI] = 0.43 to 0.68). The results of QFT-GIT and T-SPOT.TB testing at the initial LTBI screening are summarized in Table 2.

**Table 2:** Results of QuantiFERON-TB Gold In-Tube and T-SPOT.TB tests in initial evaluation of LTBI in patients with rheumatic diseases scheduled for TNF-α antagonist therapy.

	QFT-GIT			
T-SPOT.TB	Positive	Negative	Indeterminate^a^	Total
Positive	41	28	1^b^	70
Negative	4	74	2	80
Indeterminate^a^	-	6	-	6
Total	45	108	3	156

LTBI, latent tuberculosis infection; TNF, tumor necrosis factor; QFT-GIT, QuantiFERON-TB Gold In-Tube.

^a^All QFT-GIT indeterminate results occurred due to failure to generate an IFN-γ response to mitogens, and all T-SPOT.TB indeterminate results were due to excessive response of negative controls.

^b^One QFT-GIT-indeterminate patient with a 10 mm tuberculin skin test result received TB medication.

### Reversion or conversion of IGRA results in LTBI-treated patients

A total of 46 patients including 45 QFT-GIT positive patients and one QFT-GIT indeterminate patient with positive TST result received LTBI treatment. Twenty-nine patients (28 Q-T+ and one Q_I_T+) positive only by T-SPOT.TB at the initial screening did not receive LTBI treatment and proceeded to TNF-α antagonist therapy according to the study protocol (Q: QFT-GIT, T: T-SPOT.TB, +: positive, -: negative, _I_: indeterminate).

The first IGRA follow-up tests were performed after LTBI patients were treated for LTBI. The LTBI treatment regimens selected at the discretion of the treating physicians included isoniazid for 9 months, rifampin for 4 months, or both isoniazid and rifampin for 3 months. Of 46 patients who underwent LTBI treatment, two (4.3%), seven (15.2%), and 37 (80.4%) received isoniazid, rifampin, and combined isoniazid and rifampin, respectively (S1 Dataset). Four (12.1%) of 33 patients showed reversion by QFT-GIT. One patient with indeterminate QFT-GIT result at the initial screening became negative. Of 26 patients tested at the first T-SPOT.TB follow-up, none had reversion. No conversion was observed in either IGRA.

The second IGRA follow-up tests were performed in the same patient group after 263 to 1,264 days (median 445 days), during TNF-α antagonist therapy. Reversion rates were 15.8% (6/38) and 2.9% (1/34) for QFT-GIT and T-SPOT.TB, respectively, higher than the first IGRA follow-up tests. Meanwhile, two patients negative by T-SPOT.TB at the initial screening who were not tested during the first follow-up showed conversion at the second follow-up.

### Characteristics of QFT-GIT-negative/indeterminate and T-SPOT.TB-positive patients at the initial screening

We compared the clinical and laboratory characteristics of 29 patients positive only by T-SPOT.TB (28 Q-T+ and 1 Q_I_T+) with the Q+T+ patient group (Table 3). Compared to the Q+T+ patient group, the 29-patient group had a lower proportion of patients with ankylosing spondylitis (*P* value 0.047), less chance of TB treatment history (*P* value 0.04), and fewer positive TST results (*P* value <0.001). The baseline results of two IGRAs from each group are shown in Table 4. Most Q-T+ patients (26/29) had IFN-γ responses with more than 9 SFCs by T-SPOT.TB testing, while only three Q-T+ patients had 6–8 SFCs. However, the IFN-γ responses in four Q+T- patients were between 0.35–0.70 IU/mL by QFT-GIT.

**Table 3:** Comparison of patient characteristics according to baseline IGRA results.

Characteristics	QFT-GIT+ / T.SPOT TB+	QFT-GIT– or I / T.SPOT TB +	*P* value
Number of patients	41	29	
Age, years	44 (36–58)	48 (33–61)	
Number of patients >50years	16 (39)	13 (45)	0.81
Number of male patients	28 (68)	17 (59)	0.45
Diagnosis			
Rheumatoid arthritis	19 (46)	18 (62)	0.23
Ankylosing spondylitis^a^	20 (49)	7 (24)	0.047
Others ^b^	2 (5)	4 (14)	0.22
History of TB exposure	2 (5)	2 (7)	>0.999
History of TB treatment^a^	9 (22)	1 (3)	0.04
Tuberculin skin test (+)^a^	29 (71)^c^	8 (28)	<0.001
Chest X-ray (Previous TB +)	4 (10)	2 (7)	>0.999
Concurrent immunosuppressive therapy			
Methotrexate	22 (54)	18 (62)	0.62
Tacrolimus	2 (5)	1 (3)	1.00
Corticosteroid	19 (46)	20 (69)	0.09

The data are presented as median and interquartile range or as number (%).

IGRA, interferon gamma release assay; QFT-GIT, QuantiFERON-TB Gold In-Tube; +, positive; -, negative; I, indeterminate.

^a^ *P* value was less than 0.05.

^b^Ulcerative colitis, Crohn’s disease, spondyloarthropathy and psoriatic arthropathy were included.

^c^Forty patients were tested for tuberculin skin test.

**Table 4:** Results of two IGRAs stratified by baseline interferon-γ responses in different groups.

Baseline IGRA result	QFT-GIT+/T-SPOT.TB + (n = 41)	QFT-GIT-/T-SPOT.TB+ (n = 29)	QFT-GIT+/T-SPOT.TB–(n = 4)
QFT-GIT (IU/mL)			
< 0.35	0	29	0
0.35–0.70	5	0	4
0.71–1.00	6	0	0
1.01–2.99	15	0	0
> = 3.00	15	0	0
T.SPOT TB (number of spots)			
<6	0	0	4
6–8	2	3	0
9–29	6	17	0
> = 30	33	9	0

IGRA, interferon gamma release assay; QFT-GIT, QuantiFERON-TB Gold In-Tube; +, positive; -, negative; I, indeterminate.

Of 29 patients positive only by T-SPOT.TB, 19 out of 23 (83%) patients available for follow-up IGRA tests remained positive by T-SPOT.TB at a median of 482 days (range: 271–1,330 days). In particular, nine (36%) of 25 available patients showed conversion by QFT-GIT during TNF-α antagonist therapy.

### Development of active TB disease during TNF-α antagonist therapy

By the end of the follow-up period (218 to 1,264 days) in this study, four (2.9%) of 137 patients who underwent TNF-α antagonist therapy developed active TB diseases. Among them, one was Q-T+, one was Q+T-, and the remaining two were Q-T- at the initial screening. One Q-T+ and one Q+T- patient became positive by QFT-GIT and T-SPOT.TB, respectively, in follow-up tests. Two Q-T- patients were not subjected to follow-up tests. Two (50%) of patients with TB reactivation had at least one risk factor, including positive TST result or fibrotic nodules on chest radiography. The laboratory findings and clinical manifestations of these four patients are presented in Table 5.

**Table 5:** Characteristics of patients with active TB diseases during the follow-up period (218–675 days).

Patients	Sex/age	Diagnosis	ContactHistory	BCG scar	TST (mm)	Chest X-ray	IGRA changes between initial and follow-up tests		TNF	Clinical manifestations
							QFT-GIT	T-SPOT.TB		
1	F/51	RA	No	Yes	7	Fibrotic Nodules^a^	N → P	P → NT	I→E	TB pleurisy diagnosed using BAL Fluid PCR at day 218.
2	M/41	AS	No	Yes	6	N	P → P	N → P	E→I	TB peritonitis diagnosed using AFB stain and culture at day 653.
3	M/33	AS	No	Yes	10	N	N → NT	N → NT	I→E	Probable primary TB with mediastinal lymphadenitis on the chest CT at day 631.
4	F/62	RA	No	Yes	0	COPD	N → NT	N → NT	A	TB pleurisy diagnosed at day 675 from outside hospital.

TB, tuberculosis; BCG, Bacillus Calmette Guérin; TST, tuberculin skin test; IGRA, interferon-gamma release assays; QFT-GIT, QuantiFERON-TB Gold In-Tube; TNF, tumor necrosis factor; CD, Crohn’s disease; RA, rheumatoid arthritis; AS, ankylosing spondylitis; COPD, chronic obstructive pulmonary disease; N, negative; P, positive; NT, not tested; I, infliximab; E, etanercept; A, adalimumab; BAL, bronchoalveolar lavage; AFB, acid-fast bacilli.

^a^Retrospective review of initial chest radiography revealed several tiny fibrotic nodules in the right upper lobe. This finding was not recognized as an indication for LTBI treatment by the attending physician at the time of patient enrollment.

## Discussion

TNF-α antagonists are currently used to treat patients with various autoimmune diseases in gastroenterology, rheumatology and dermatology. TNF-α antagonists are generally considered to have a favorable benefit/risk ratio [12], and considered indispensable for treatment of patients with rheumatic diseases. However, adverse effects such as TB reactivation and development of other infections or lymphomas have been associated with use of these biologics. Therefore, prior to initiating TNF-α antagonist therapy, patients should be tested to avoid these possible side effects. In this study, we investigated two different IGRAs as potentially superior ways of diagnosing LTBI in patients with rheumatic diseases before and during TNF-α antagonist therapy.

According to our current protocol using QFT-GIT with supplementary TST results, the prevalence of LTBI patients with rheumatic diseases before starting TNF-α antagonist therapy was 29.5% (46/156) at the initial screening. This result is comparable to the LTBI prevalence of 35% in a different cohort previously reported by our group [18]. Similarly, the Korean National Guidelines [14, 15] estimated the LTBI prevalence to be 33% in 2004. The prevalence of LTBI rose to 44.9% (70/156) in our patient group based on T-SPOT.TB test results. The positive rates for both QFT-GIT and T-SPOT.TB in this study were higher than those of other studies reported from other countries [19, 20]. Mínguez et al. [19] reported 17% and 20.8% QFT-GIT and T-SPOT.TB positive rates, respectively, among 53 rheumatic patients in Spain. Vassilopoulos et al. [20] presented positive rates of 21% and 25% for QFT-GIT and T-SPOT.TB, respectively, among 155 patients in Greece. This difference in prevalence may be explained by higher TB incidence in Korea compared to Spain and Greece.

After LTBI treatment and long-term follow-up visits, the reversion rates of QFT-GIT were 12.1% (4/33) and 15.8% (6/38), respectively. The reversion rates might be partially attributed to LTBI treatment, since previous studies [21–23] have reported that LTBI treatment could result in a gradual decrease of IFN-γ response. Reversion may also be spontaneous, as IGRA instability and variability have been shown in several studies [6, 24]. Spontaneous reversions are likely to occur in subjects with borderline IGRA results, and even more likely in TST-negative cases [25, 26]. However, we should be cautious in applying the latter explanation to our results because except for four patients, all patients who received LTBI treatment had positive IFN-γ responses by both IGRAs. Those four patients had QFT-GIT IFN-γ responses between 0.35–0.70, which is a borderline zone for interpretation. Kim et al. [9] reported an IGRA reversion rate of 4.5% (3/66) among 66 patients with diverse rheumatic diseases, using two IGRAs (QuantiFERON-TB Gold and QuantiFERON-TB Gold In-Tube), much lower than the reversion rates observed in our study. Differences in patient groups and test methods might explain this discrepancy.

Twenty-nine patients were positive only by T-SPOT.TB at the initial screening. Based on published meta-analyses, the sensitivity of this test appears to be higher than that of QFT-GIT and TST [27, 28]. When considering the intermediate TB prevalence in Korea, the increased sensitivity of the T-SPOT.TB test may have an advantage over QFT-GIT, especially in patients at high risk of LTBI. Therefore, negative QFT-GIT results in patients positive by T-SPOT.TB should be interpreted with caution, as there is a possibility for false negative findings. It might be helpful to use T-SPOT.TB along with QFT-GIT in the initial assessment and during the follow-up period in patients at high risk of LTBI.

Comparison of the 29 patients positive only by T-SPOT.TB (28 Q-T+ and one Q_I_T+) with Q+T+ 41-patient group revealed that the 29-patient group had a lower chance of TB treatment history and positive TST results. This result may suggest that the T.SPOT TB test should be interpreted carefully with the assumption that it could be more independent from other TB risk factors such as past history of TB, BCG vaccination, or TST results when compared with QFT-GIT. Further studies with sound evidence are necessary to prove this argument.

Of note, four patients had indeterminate QFT-GIT results during the study period. These indeterminate results all occurred due to failure to generate an IFN-γ response to mitogens, and all four patients were administered corticosteroid treatments (S1 Dataset). Since several studies [29–31] indicated that steroid use is associated with a high likelihood of indeterminate QFT-GIT results due to mitogen anergy, the indeterminate results in our study might have been caused by the immunosuppressive effect of corticosteroids in the same context. We previously reported [32] that incubation delay could lead to indeterminate QFT-GIT results. However, because incubation occurred within 6 hours of collection and the rate of indeterminate results has consistently been below 5% at our institute, incubation delays were not likely to affect the results of this study.

Seventy-five (48%) patients with rheumatoid arthritis were included in our study. A recent TBNET study by Sester et al. [28] compared the performance of TST, T-SPOT.TB and QFT-GIT in immunocompromised individuals, including patients with rheumatoid arthritis (n = 111). In their study, the frequency of positive test results in rheumatoid arthritis patients was highest for TST (37.2%), and lowest for QFT-GIT (25.0%), whereas in our study, the highest and lowest positivity rates in rheumatoid arthritis patients were T-SPOT.TB (49.3%) and TST (25.3%). There was substantial agreement between T-SPOT.TB and QFT-GIT results (κ-value = 0.77) in the rheumatoid arthritis patient group in the TBNET study, while the agreement was moderate (κ-value = 0.46) in our study (S1 Dataset). These differences might be partly due to different TB epidemiology and differences in background of study subjects between these studies. The TBNET was a multicenter study based in Europe, where TB incidence is generally low, and most of the study subjects with rheumatoid arthritis were white (96.5%).

Four patients (2.9%) experienced LTBI reactivation during TNF-α antagonist therapy. Two (2/4) were positive either by QFT-GIT or T-SPOT.TB at initial screening, and later one patient became positive by both IGRAs during follow-up tests, and another patient showed conversion by QFT-GIT but was not tested by T-SPOT.TB. The remaining two (2/4) patients were Q-T- at the initial screening and no further IGRAs were performed. In addition, two of four patients with LTBI reactivation had at least one TB risk factor, including abnormal chest radiography findings consistent with previous TB infection (1/2), and positive TST results (1/2). Four patients with LTBI reactivation were observed at a median of 642 days (range: 218 to 675 days), which was a relatively long period. Therefore, we could not definitely determine whether those four patients had genuine LTBI reactivation or were newly exposed to TB. New TB infections were especially possible for the two Q-T- patients. The other two patients were positive for at least one of the IGRAs at initial screening and were thus more likely to have experienced LTBI reactivation.

Previous global clinical studies [1, 33–35] suggested that the TB reactivation rate during TNF-α antagonist therapy was approximately 0.01%, and might be higher in TB- endemic countries. Similarly, a previous study [18] from our group of 107 Korean patients with either rheumatoid arthritis or ankylosing spondylitis who received TNF-α antagonist therapy and were followed up for 13–33.5 months found that no patients developed active TB. In this present study, however, 2.9% (4/137) of patients developed active TB while receiving TNF-α antagonist therapy, a relatively higher rate than the previous reports. Given that four patients had TB reactivation over the course of 7–22 months, it could be inferred that LTBI follow-up over longer periods might be necessary. Since we observed patients for a median of 13.6 months, the optimal follow-up period remains to be determined in future studies

Until now, there has been neither a single gold standard test nor clear-cut guideline for LTBI diagnosis. It has been a challenge to determine the usefulness of IGRAs for LTBI monitoring during TNF-α antagonist therapy. Several studies [8, 19, 20, 36, 37] have evaluated IGRAs for LTBI screening in these patients, suggesting that QFT-GIT, T-SPOT.TB, and TST are all acceptable, but imperfect tests for definitive LTBI diagnosis. Based on the results of our study, we suggest that T-SPOT.TB test should be included in the initial evaluation as well as in follow-up protocols for patients with rheumatic diseases receiving TNF-α antagonist therapy. Its increased sensitivity over QFT-GIT and TST might increase LTBI detection rates.

The observation of two patients with LTBI reactivation in this study who were negative by both IGRAs at initial screening suggested the importance of follow-up LTBI screening for all patients, including those with negative IGRA results in baseline screening. Also, any change that might possibly be a sign of TB infection or disease, including TST or IGRA conversion and chest radiography alteration during the follow-up period needs to be investigated carefully in all patients at high risk of LTBI.

One significant issue in this study was that the number of enrolled patients was not large enough to generalize the frequency of TB reactivation in patients undergoing TNF-α antagonist therapy. Larger prospective studies are necessary to better understand not only TB reactivation rates, but also IGRA reversion and conversion in patients with rheumatic diseases during TNF-α antagonist therapy. It also should be noted that our patients were all Koreans living in a country with a moderate TB-burden. Therefore, the results of this study may not apply to other ethnic groups in regions with different TB epidemiologies.

This study demonstrates the need to consider both QFT-GIT and T-SPOT.TB for more sensitive diagnosis of LTBI in patients with rheumatic diseases, especially those with at least one risk factor for TB, such as abnormal chest radiography findings consistent with previous TB infection, positive TST result, and TB contact history.

In conclusion, it would be beneficial to perform both QFT-GIT and T-SPOT.TB as main diagnostic tools with supplementary TST in vulnerable patients not only at the initial assessment but also during the follow-up period. This would reduce the number of patients with LTBI reactivation by facilitating timely and accurate LTBI diagnosis. Clinical information of patients should also be considered when determining whether to start preventive LTBI treatment, when to do follow-up tests, and how to interpret test results.

## Supporting Information

S1 Dataset Case report form containing all patients’ clinical information and test results. (XLS)
